# Supplementary material for: Identification of photoperiod-induced specific miRNAs in the adrenal glands of Sunite sheep (Ovis aries)
Source: Front Vet Sci. 2022 Jul 22;9:888207. doi: 10.3389/fvets.2022.888207 (PMC9354845; doi:10.3389/fvets.2022.888207)
Supplement: Supplementary file 1 [file Data_Sheet_1.ZIP › Supplementary file/miRDeep2 file.zip!/miRDeep2 file/Know_Structure/SPLP42A2/oar-mir-494.pdf]

miRBase precursor : oar-mir-494
Total read count : 14997
oar-miR-494-5p read count : 62
oar-miR-494-3p read count : 14931
remaining reads : 4

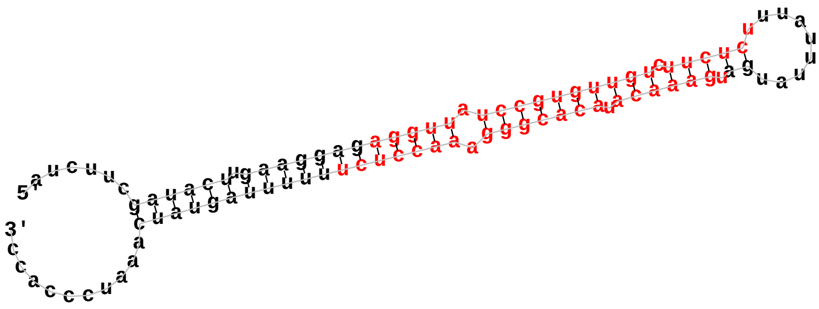

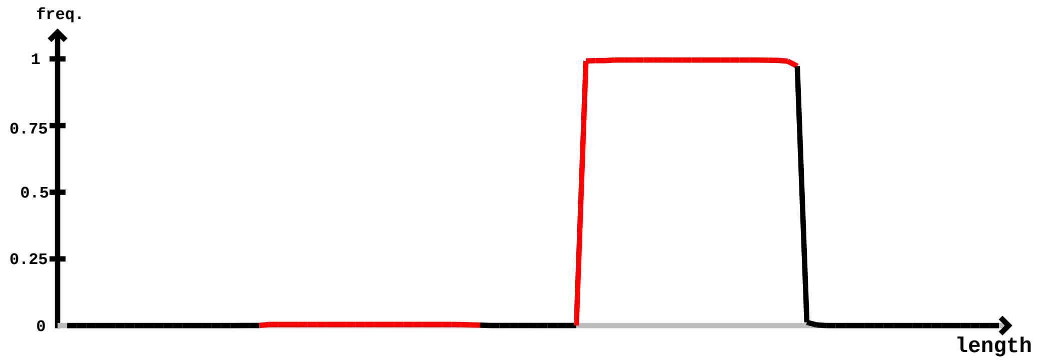


oar-miR-494-3p

oar-miR-494-5p

```
5'- aucuucgauacuugaaggagagguuauccguguugucuucucuuuauuuaugaugaaacauacacgggaaaccucuuuuuuaguaucaaaucccacc  -3'    exp
    ......((((((.((((((((((((.((((((((((.(((((.........)).)))))).))))))).))))))))))))))))))..........   reads   mm   sample
    .................gagagguuauccguguugucuu..........................................................   1       0    seq
    ..................agagguuauccguguugucuu..........................................................   1       0    seq
    ..................agagguuauccguguugucuuc.........................................................   3       0    seq
    ....................agguuauccguguugucuucu........................................................   17      0    seq
    ....................agguuauccguguugucuucuc.......................................................   16      0    seq
    ....................agguuauccguguugucuucucu......................................................   20      0    seq
    ....................agguuauccguguugucuucucuu.....................................................   2       0    seq
    ....................agguuauccguguugucuucucuuu....................................................   2       0    seq
    ..........................uccguguugucuucucuuuauu.................................................   1       0    seq
    .............................................auuuaugaugaaacaua...................................   1       0    seq
    .............................................auuuaugaugaGacaua...................................   2       1    seq
    ....................................................augaaacauacacgggaaaccucu.....................   2       0    seq
    .....................................................ugaaacauacacgggaaa..........................   1       0    seq
    .....................................................ugaaacauacacgggaaac.........................   10      0    seq
    .....................................................ugaaacauacacgggaaacc........................   7       0    seq
    .....................................................ugaaacauacacgggaaaccu.......................   45      0    seq
    .....................................................ugaaacauacacgggaaaccuc......................   281     0    seq
    .....................................................ugaaacauacacgggaaaccucu.....................   14382   0    seq
    .....................................................ugaaacauacacgggaaaccucuu....................   142     0    seq
    .....................................................ugaaacauacacgggaaaccucuuu...................   7       0    seq
    ......................................................gaaacauacacgggaaaccucu.....................   20      0    seq
    .......................................................aaacauacacgggaaaccucu.....................   1       0    seq
    ........................................................aacauacacgggaaaccucuu....................   1       0    seq
    ........................................................aacauacacgggaaaccucuuu...................   30      0    seq
    ........................................................aacauacacgggaaaccucuuuu..................   1       0    seq
    ...........................................................auacacgggaaaccucu.....................   1       0    seq
```
